# Cysteine Peptidases, Secreted by *Trichomonas gallinae*, Are Involved in the Cytopathogenic Effects on a Permanent Chicken Liver Cell Culture

**DOI:** 10.1371/journal.pone.0037417

**Published:** 2012-05-23

**Authors:** Aziza Amin, Katharina Nöbauer, Martina Patzl, Evelyn Berger, Michael Hess, Ivana Bilic

**Affiliations:** 1 Clinic for Avian, Reptile and Fish Medicine, Department for Farm Animals and Veterinary Public Health, University of Veterinary Medicine, Vienna, Austria; 2 VetCore Facility for Research, University of Veterinary Medicine, Vienna, Austria; 3 Clinical Immunology, Department for Pathobiology, University of Veterinary Medicine, Vienna, Austria; Stanford University, United States of America

## Abstract

*Trichomonas gallinae*, the aetiological agent of avian trichomonosis, was shown to secrete soluble factors involved in cytopathogenic effect on a permanent chicken liver (LMH) cell culture. The present study focused on the characterization of these molecules. The addition of specific peptidase inhibitors to the cell-free filtrate partially inhibited the monolayer destruction, which implied the presence of peptidases in the filtrate and their involvement in the cytopathogenic effect. One-dimensional substrate (gelatin) SDS-PAGE confirmed the proteolytic character of the filtrate by demonstrating the proteolytic activity within the molecular weight range from 38 to 110 kDa. In addition, the proteolytic activity was specifically inhibited by addition of TLCK and E-64 cysteine peptidase inhibitors implying their cysteine peptidase nature. Furthermore, variations in the intensity and the number of proteolytic bands were observed between cell-free filtrates of low and high passages of the same *T. gallinae* clonal culture. Two-dimensional substrate gel electrophoresis of concentrated *T. gallinae* cell-free filtrate identified at least six proteolytic spots. The mass spectrometric analysis of spots from 2-D gels identified the presence of at least two different Clan CA, family C1, cathepsin L-like cysteine peptidases in the cell-free filtrate of *T. gallinae*. In parallel, a PCR approach using degenerated primers based on the conserved amino acid sequence region of cysteine peptidases from *Trichomonas vaginalis* identified the coding sequences for four different Clan CA, family C1, cathepsin L-like cysteine peptidases. Finally, this is the first report analyzing molecules secreted by *T. gallinae* and demonstrating the ubiquity of peptidases secreted by this protozoon.

## Introduction

*Trichomonas gallinae*, a flagellated protozoon is commonly found in the upper digestive tract of different bird species, including columbid, passerine, and psittacine birds as well as falconiformes [1]–[6], but can also affect other organs depending on the virulence of the strain [7]. The domestic pigeon (*Columba livia*) is the primary host of the flagellate which has been considered responsible for the worldwide spread of the parasite [8]. With its global distribution *T. gallinae* is the causative agent of avian trichomonosis causing serious losses also in wild birds, in particular in wild finches where several outbreaks were noticed [9]–[15]. Studies with *T. gallinae* demonstrated a wide spectrum of virulence, ranging from virulent strains to avirulent ones [9], whereas the molecular investigations demonstrated genetic diversity between different strains of this parasite [16]–[18].

Numerous investigations reported the interaction of *T. gallinae* with various hosts [2], [7], [19]–[23], but only a few demonstrated the behaviour of this parasite in cell cultures [24]–[26]. We recently demonstrated that genetically different *T. gallinae* isolates caused diverse magnitude of a cytopathogenic effect on permanent chicken liver cell (LMH) and permanent quail fibroblast (QT35) monolayers [25]. In contrast to other studies which focused on the interaction of *T. gallinae* with cell cultures, Amin et al. [25] demonstrated that the observed destruction of monolayers was the consequence of both direct and indirect interaction of cell cultures and the parasite. Cytopathogenic changes in tissue cultures observed upon the exposure of cells to substances released by the parasite into the culture media were also reported for *T. vaginalis*, a close relative of *T. gallinae* [27]–[30]. Extensive research performed on the analysis of the *T. vaginalis* culture media revealed the presence of various cysteine peptidases and other molecules that mediate cytotoxicity by damaging the target cell plasma membrane reviewed in Schwebke and Burgess [31]. Some of these cytotoxic molecules have perforin-like activity and create pores in erythrocyte membranes reviewed in Fiori et al. [32]; whereas others are different lytic factors with phospholipase A_2_ activities to destroy nucleated cells and erythrocytes [33].

Cysteine peptidases play essential roles in biology and pathogenicity of different parasites, reviewed in Sajid and McKerrow [34]. In the case of *T. vaginalis*, cysteine peptidases have been implicated in the virulence [35]–[37], the cytotoxicity [29], the adherence to host cells [35], [36], [38], [39] and the detachment of host cells [40]. Furthermore, their action was associated with nutrient acquisition [37], [41], hemolysis [42] and the evasion of the host immune response [43]–[45].

The present study focused on the molecular characterization of cell-free filtrates from *T. gallinae* axenic cultures, which were previously shown to possess cytopathogenic effects on permanent chicken liver (LMH) cells. The identification of Clan CA, family C1, cathepsin L-like cysteine peptidases in the cell-free filtrate and demonstration of their involvement in the cytopathogenic effects of the filtrate suggest the virulent role of these peptidases in the pathogenesis of *T. gallinae*.

## Results

### Influence of peptidase inhibitors on the cytopathogenic effect of cell-free filtrate from axenically grown *T. gallinae*

**Figure 1:**
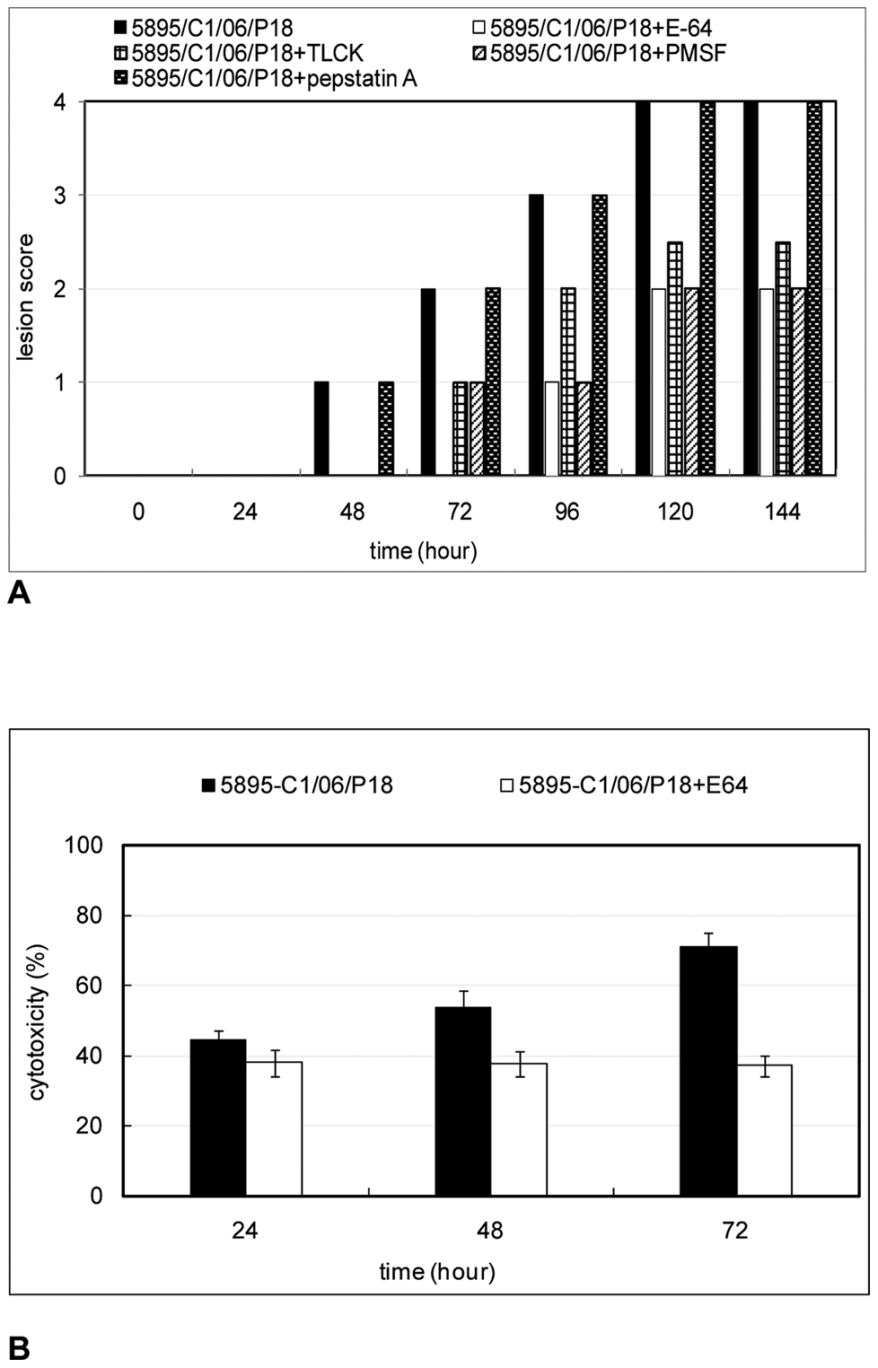
Influence of peptidase inhibitors on the cytopathogenic effect of the cell-free filtrate. LMH monolayer was incubated with cell-free filtrate with and without different peptidase inhibitors. (A) Mean lesion scores, (B) cytotoxicity of LMH cells, as assessed by Promega CellTiter 96® aqueous solution at different time points. Cell-free filtrate was obtained after 24 h of incubation of 10^7^ axenically grown protozoa cells obtained from *T. gallinae* clone 5895-C1/06, passage18. Absorbance values for Promega CellTiter 96® aqueous solution were recorded at 490 nm using ELISA reader.

In order to establish the maximal concentration of each peptidase inhibitor without toxicity for the monolayer, incubations with different concentrations were performed. Accordingly, the suitable concentration for each inhibitor was used in this study. The results showed that the addition of either 1 mM PMSF, 270 μM E-64 or 135 μM TLCK to the cell-free filtrate of *T. gallinae* partially inhibited cytopathogenic effects induced by trichomonads (Figure 1). The inhibition was assayed by the detachment of the monolayer in comparison to the filtrate without inhibitors (Figure 1A). After applying peptidase inhibitors the monolayer destruction was reduced, but differences between the actions of these inhibitors were noticed. The E-64, cysteine peptidase inhibitor, produced the best inhibition causing the lowest destruction of the monolayer. The application of Pepstatin A (5 μM), aspartic peptidase inhibitor, had no inhibitory effect on the filtrate (Figure 1A).

The effect of the E-64, cysteine peptidase inhibitor, on the cell-free filtrate was also assayed by CellTiter 96® aqueous one solution cell proliferation assay and showed the significant reduction in cytotoxicity (Figure 1B). After 72 h of incubation the maximum cytotoxicity of *T. gallinae* clone 5895-C1/06, passage 18, on LMH cells, produced by the cell-free filtrate without E-64, was 70.9%. In the presence of the inhibitor the toxicity of the filtrate could be reduced to 37.1%.

### Demonstration of peptidase activity in *T. gallinae* cell-free filtrates using one-dimensional substrate gel electrophoresis

**Figure 2:**
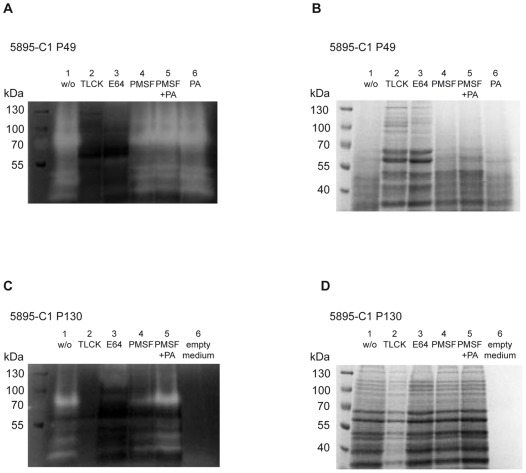
Demonstration of protease activity in cell-free filtrates from *T. gallinae* clone 5895-C1/06 by one-dimensional substrate SDS-PAGE. A) Substrate (0.2% gelatin) 1-D SDS-PAGE (8%) with the low passage (49x) cell-free filtrate, B) conventional 1-D SDS PAGE (8%) with the low passage (49x) cell-free filtrate, C) Substrate (0.2% gelatin) 1-D SDS-PAGE (8%) with the high passage (130x) cell-free filtrate, and D) conventional 1-D SDS PAGE (8%) with the high passage (130x) cell-free filtrate.

In order to detect whether *T. gallinae* cells secreted peptidases into trichomonad-growth medium (HF medium), concentrated cell-free filtrates from clone 5895-C1/06 with and without peptidase inhibitors were separated by SDS-PAGE with gelatin copolymerized as substrate (Figure 2). Additionally, filtrates of lower (P49) and high passages (P130) from clone 5895-C1/06 were compared. The zymogram of the cell-free filtrate from P49 of clone 5895-C1/06 without peptidase inhibitors showed a proteolytic region of at least five different clear bands with molecular weights of approximately 38, 41, 50, 80 and 110 kDa (Figure 2A). In contrary, the zymogram analysis of the cell-free filtrates from P130 of clone 5895-C1/06 demonstrated weaker proteolytic activity (Figure 2C). The clear bands indicative for proteolytic activity were of weaker intensity and the 110 kDa band present in zymogram of cell-free filtrate from P49 was absent. No proteolytic bands were observed on both zymograms of cell-free filtrates containing peptidase inhibitors TLCK (cysteine and some serine peptidases) and E-64 (cysteine peptidases) (Figure 2A, 2C). Samples treated with PMSF (serine peptidase inhibitor) and Pepstatin A demonstrated the same proteolytic regions as the sample without inhibitors (Figure 2A, 2C). In parallel, the same samples were investigated by conventional SDS-PAGE (Figure 2B and 2D). Cell-free filtrate from clone 5895-C1/06 P49 containing TLCK and E-64 produced a pattern of sharp bands of different molecular weights (Figure 2B). In contrary, in samples without inhibitors or those with PMSF and Pepstatin A, this pattern was not prominent and the remaining bands were not sharp. This additionally confirmed the presence of peptidase activity in the cell-free filtrate of P49 of clone 5895-C1/06 which could be specifically blocked by addition of TLCK and E-64. Interestingly, conventional SDS-PAGE of filtrates from P130 of clone 5895-C1/06 demonstrated sharp bands in all samples even in the ones without peptidase inhibitors (Figure 2D).

### Detection of Clan CA, family C1, cathepsin L-like cysteine peptidases in *T. gallinae* cell-free filtrate by MS analysis of peptidase spots from 2-D gel

**Figure 3:**
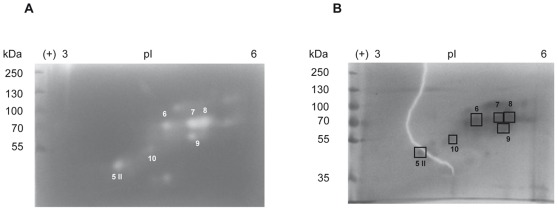
Two-dimensional gel electrophoresis of the cell-free filtrate from clone 5895-C1/06, P53. A) Substrate (0.2% gelatine) SDS-PAGE (8%) and B) conventional SDS-PAGE (8%) was used as the second dimension, respectively.

In order to further characterize the proteolytic activity of the cell-free filtrate from *T. gallinae*, the concentrated proteins of cell free-filtrates from passage 53 of clone 5895-C1/06 were investigated by conventional and substrate 2-D gel electrophoresis (Figure 3). Zymographic analysis identified at least seven spots with proteolytic activity between 35 and 100 kDa, which displayed p*I* values between 3.7 and 5.3 (calculated according to linear distribution of pH across the strip). The MS analysis of six spots of conventional 2-D gels, which corresponded to the proteolytic spots found in 2-D zymograms, identified the presence of Clan CA, family C1 and cathepsin L-like cysteine peptidases in cell-free filtrate of *T. gallinae*. In five proteolytic spots (6, 7, 8, 9, 10) a peptide with the same mass (m/z 1466.6) was identified (Figure S1). Manual peptide de Novo sequencing of this charged peptide identified the following sequence: FSY [I/L]ADYPYTAR (Figure S2). The MS homology search matched this peptide to different Clan CA, family C1, cathepsin L-like cysteine peptidases of *T. vaginalis* (Table 1). In spot 8 (70 kDa, p*I* 5.2) two additional peptides with masses of m/z 850 and m/z 1121 could be identified (Figure S1). Manual sequencing of the tandem mass spectra corresponding to the single peptides identified following peptide sequences: NYW [I/L]VR (m/z 850.5) and NSWGASWGEK (m/z 1121.4) (Figure S2). The MS homology search using all three peptides matched these peptides with highest homology to *T. vaginalis* Clan CA, family C1, cathepsin L-like cysteine peptidase TVAG_355480 (accession number XP_001310117) (Table 2).

**Table 1:** Results of the MS homology search for the peptide m/z 1466.

Protein Score	Peptide Score	Peptide Sequence	Matching Sequence	MS-Digest Index Nr.	Protein MW (Da)/pI	Accession Nr.	Species	Protein Name
48	48	FSYIADYPYTAR	(K)F**MLT**ADYPYTAR(D)	**2595889**	**34641/8.4**	**Q27107**	*T. vaginalis*	Clan CA, family C1, cathepsin L-like cysteine peptidase
	48	FSYLADYPYTAR	(K)F**MLT**ADYPYTAR(D)					
45	45	FSYIADYPYTAR	(K)F**MTE**ADYPYTAR(D)	**1626529**	**33768/7.5**	**A2E0N7**	*T. vaginalis*	Clan CA, family C1, cathepsin L-like cysteine peptidase
	45	FSYLADYPYTAR	(K)F**MTE**ADYPYTAR(D)					
45	45	FSYIADYPYTAR	(K)F**MTE**ADYPYTAR(D)	**1641664**	**33796/6.9**	**A2FD35**	*T. vaginalis*	Clan CA, family C1, cathepsin L-like cysteine peptidase
	45	FSYLADYPYTAR	(K)F**MTE**ADYPYTAR(D)					
45	45	FSYIADYPYTAR	(K)F**MTE**ADYPYTAR(D)	**2595891**	**32281/7.6**	**Q27109**	*T. vaginalis*	Cysteine proteinase, putative (Fragment)
	45	FSYLADYPYTAR	(K)F**MTE**ADYPYTAR(D)					
45	45	FSYIADYPYTAR	(K)F**MTE**ADYPYTAR(D)	**2632915**	**33798/7.5**	**Q49P75**	*T. vaginalis*	Cathepsin L-like cysteine proteinase
	45	FSYLADYPYTAR	(K)F**MTE**ADYPYTAR(D)					

All matches selected by MS homology search are displayed. Following parameters were used for search: Taxonomy search in *Trichomonas vaginalis*; Database searched: UniProtKB.2011.01.11; Mass Tolerance: 0.5 Da; Digest Used: No enzyme; Max. # Missed Cleavages: 1; Min matches: 1; Score matrix: BLOSUM62; Peptide Sequence: 1 FSY [I|L]ADYPYTAR 3 (number left to the peptide sequence is used as the name of the peptide, whereas the number right to the peptide sequence refers to the maximal number of allowed mismatches). Mismatched amino acids are shown in bold.

**Table 2:** Results of the MS homology search for the protein Spot 8.

Protein Score	Peptide Score	Peptide Sequence	Matching Sequence	MS-Digest Index Nr.	Protein MW (Da)/pI	Accession Nr.	Species	Protein Name
140	45	FSYIADYPYTAR	(K)F**MTE**ADYPYTAR(D)	**1641664**	**33796/6.9**	**A2FD35**	*T. vaginalis*	Clan CA, family C1, cathepsin L-like cysteine peptidase
	45	FSYLADYPYTAR	(K)F**MTE**ADYPYTAR(D)					
	58	NSWGASWGEK	(R)NSWG**T**SWGEK(G)					
	37	NYWIVR	(K)NYWIVR(N)					
139	48	FSYIADYPYTAR	(K)F**MLT**ADYPYTAR(D)	**2595889**	**34641/8.4**	**Q27107**	*T. vaginalis*	Clan CA, family C1, cathepsin L-like cysteine peptidase
	48	FSYLADYPYTAR	(K)F**MLT**ADYPYTAR(D)					
	54	NSWGASWGEK	(R)NSWG**T**SWGEQ(G)					
	37	NYWIVR	(K)NYWIVR(N)					
137	45	FSYIADYPYTAR	(K)F**MTE**ADYPYTAR(D)	**1626529**	**33768/7.5**	**A2E0N7**	*T. vaginalis*	Clan CA, family C1, cathepsin L-like cysteine peptidase
	45	FSYLADYPYTAR	(K)F**MTE**ADYPYTAR(D)					
	55	NSWGASWGEK	(R)NSWG**TA**WGEK(G)					
	37	NYWIVR	(K)NYWIVR(N)					
137	45	FSYIADYPYTAR	(K)F**MTE**ADYPYTAR(D)	**2595891**	**32281/7.6**	**Q27109**	*T. vaginalis*	Cysteine proteinase, putative (Fragment)
	45	FSYLADYPYTAR	(K)F**MTE**ADYPYTAR(D)					
	55	NSWGASWGEK	(R)NSWG**TA**WGEK(G)					
	37	NYWIVR	(K)NYWIVR(N)					
137	45	FSYIADYPYTAR	(K)F**MTE**ADYPYTAR(D)	**2632915**	**33798/7.5**	**Q49P75**	*T. vaginalis*	Cathepsin L-like cysteine proteinase
	45	FSYLADYPYTAR	(K)F**MTE**ADYPYTAR(D)					
	55	NSWGASWGEK	(R)NSWG**TT**WGEK(G)					
	37	NYWIVR	(K)NYWIVR(N)					
99	62	NSWGASWGEK	(R)NSWGASWGEK(G)	**1641445**	**33981/5.7**	**A2FCE4**	*T. vaginalis*	Clan CA, family C1, cathepsin L-like cysteine peptidase
	37	NYWIVR	(A)NYWIVR(N)					
95	58	NSWGASWGEK	(R)NSWG**T**SWGEK(G)	**1635330**	**33644/6.2**	**A2ET02**	*T. vaginalis*	Clan CA, family C1, cathepsin L-like cysteine peptidase
	37	NYWIVR	(K)NYWIVR(N)					
95	58	NSWGASWGEK	(R)NSWG**T**SWGEK(G)	**2044283**	**33663/6.3**	**B6CAS9**	*T. vaginalis*	Cytotoxic cysteine proteinase
	37	NYWIVR	(K)NYWIVR(N)					
95	58	NSWGASWGEK	(R)NSWG**V**SWGEK(G)	**2595888**	**34408/6.7**	**Q27106**	*T. vaginalis*	Clan CA, family C1, cathepsin L-like cysteine peptidase
	37	NYWIVR	(K)NYWIVR(N)					
94	57	NSWGASWGEK	(R)NSWG**E**SWGEK(G)	**1650317**	**31394/7.9**	**A2G6Q5**	*T. vaginalis*	Clan CA, family C1, cathepsin L or K-like cysteine peptidase
	37	NYWIVR	(K)NYWIVR(N)					

The top 10 matches from the search are displayed. Following parameters were used for search: taxonomy search in *Trichomonas vaginalis*; Database searched: UniProtKB.2011.01.11; Mass Tolerance: 0.5 Da; Digest Used: No enzyme; Max. # Missed Cleavages: 1; Min matches: 1; Score matrix: BLOSUM62; List of Peptide Sequences: 1 FSY [I|L]ADYPYTAR 3, 2 NYW [I|L]VR 1, 3 NSWGASWGEK 2 (number left to the peptide sequence is used as the name of the peptide, whereas the number right to the peptide sequence refers to the maximal number of allowed mismatches). Mismatched amino acids are shown in bold.

The MS/MS spectrum of the sixth proteolytic spot, protein spot 5II (45 kDa, p*I* 3.7), identified two peptides with masses of m/z 850.5 and m/z 1879.8 (Figure S1). The manual peptide de Novo sequencing identified peptides with the sequences: NYWI/LVR (m/z 850) and VNVVEGDEADLATK (m/z 1879) (Figure S3). The MS homology search using either combination of NYWI/LVR and VNVVEGDEADLATK matched these peptides with the highest homology to *T. vaginalis* Clan CA, family C1, cathepsin L-like cysteine peptidase TVAG_298080 (accession number XP_001316414) and *T. vaginalis* CP39-Cytotoxic cysteine proteinase (accession number ABX56032) (Table 3).

**Table 3:** Results of the MS homology search for the protein Spot 5II.

Protein Score	Peptide Score	Peptide Sequence	Matching Sequence	MS-Digest Index Nr.	Protein MW (Da)/pI	Accession Nr.	Species	Protein Name
100	37	NYWIVR	(K)NYWIVR(N)	**1635330**	**33644/6.2**	**A2ET02**	*T. vaginalis*	Clan CA, family C1, cathepsin L-like cysteine peptidase
	63	VNVVEGDEADLATK	(Y)VNVVEGDE**K**DLATK(V)					
100	37	NYWIVR	(K)NYWIVR(N)	**2044283**	**33663/6.3**	**B6CAS9**	*T. vaginalis*	Cytotoxic cysteine proteinase
	63	VNVVEGDEADLATK	(Y)VNVVEGDE**K**DLATK(V)					
96	37	NYWIVR	(K)NYWIVR(N)	**1626529**	**33768/7.5**	**A2E0N7**	*T. vaginalis*	Clan CA, family C1, cathepsin L-like cysteine peptidase
	59	VNVVEGDEADLATK	(Y)VNV**A**EGDE**K**DLATK(V)					
96	37	NYWIVR	(K)NYWIVR(N)	**1641664**	**33796/6.9**	**A2FD35**	*T. vaginalis*	Clan CA, family C1, cathepsin L-like cysteine peptidase
	59	VNVVEGDEADLATK	(Y)VNV**A**EGDE**K**DLATK(V)					
96	37	NYWIVR	(K)NYWIVR(N)	**2595891**	**32281/7.6**	**Q27109**	*T. vaginalis*	Cysteine proteinase, putative (Fragment)
	59	VNVVEGDEADLATK	(Y)VNV**A**EGDE**K**DLATK(V)					
96	37	NYWIVR	(K)NYWIVR(N)	**2632915**	**33798/7.5**	**Q49P75**	*T. vaginalis*	Cathepsin L-like cysteine proteinase
	59	VNVVEGDEADLATK	(Y)VNV**A**EGDE**K**DLATK(V)					
94	37	NYWIVR	(K)NYWIVR(N)	**1621053**	**33733/7.0**	**A2DJ07**	*T. vaginalis*	Clan CA, family C1, cathepsin L-like cysteine peptidase
	57	VNVVEGDEADLATK	(Y)**I**NVVEGDE**K**DLAAK(V)					
94	37	NYWIVR	(K)NYWIVR(N)	**1636066**	**34189/6.8**	**A2EVA2**	*T. vaginalis*	Clan CA, family C1, cathepsin L-like cysteine peptidase
	57	VNVVEGDEADLATK	(Y)**I**NVVEGDE**K**DLAAK(V)					
92	31	NYWIVR	(T)**K**YWIVR(N)	**1638461**	**33928/5.1**	**A2F2X0**	*T. vaginalis*	Clan CA, family C1, cathepsin L-like cysteine peptidase
	61	VNVVEGDEADLATK	(Y)**I**NVVEGDENDLATK(I)					
81	37	NYWIVR	(K)NYWIVR(N)	**1650317**	**31394/7.9**	**A2G6Q5**	*T. vaginalis*	Clan CA, family C1, cathepsin L or K-like cysteine peptidase
	44	VNVVEGDEADLATK	(Y)V**T**VNEGDE**K**DLA**K**K(V)					
81	37	NYWIVR	(K)NYWIVR(N)	**2761380**	**31250/6.9**	**Q6UEJ4**	*T. vaginalis*	Papain-like cysteine proteinase
	44	VNVVEGDEADLATK	(Y)V**T**VNEGDE**K**DLA**K**K(V)					

The top 10 matches from the search are displayed. Following parameters were used for search: Taxonomy search in *Trichomonas vaginalis*; Database searched: UniProtKB.2011.01.11; Mass Tolerance: 0.5 Da; Digest Used: No enzyme; Max. # Missed Cleavages: 1; Min matches: 1; Score matrix: BLOSUM62; List of Peptide Sequences: 1 VNVVEGDEADLAT [Q|K] 4, 2 NYW [I|L]VR 1 (number left to the peptide sequence is used as the name of the peptide, whereas the number right to the peptide sequence refers to the maximal number of allowed mismatches). Mismatched amino acids are shown in bold.

### Determination of cysteine peptidase coding sequences

**Figure 4:**
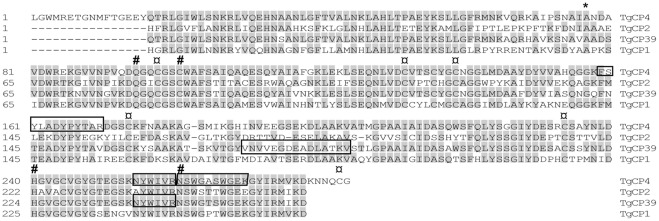
Comparison of the predicted amino acid sequences of *T. gallinae* cysteine peptidases. Residues identical to consensus are shaded grey; * above a residue indicates a predicted start of mature protein; # above a residue indicates key active site; conserved cysteine residues involved in formation of disulphide bonds are labelled with.

In order to determine sequences that encode for cysteine peptidases detected by MS-analysis, a PCR approach using degenerate primers was applied. Since the genomic sequence of *T. gallinae* is unknown, the design of primers was based on the conserved amino acid sequences of *T. vaginalis* cysteine peptidases. Sequence analyses of PCR products revealed four different genes encoding cysteine peptidases, named: TgCP1, TgCP2, TgCP4, and TgCP39. The names were given according to the homologues present in *T. vaginalis*. Sequences reported here are not full gene sequences, since the 5′- and 3′- untranslated regions were not determined. The predicted proteins of all four determined gene sequences display most of the features typical for Clan CA, family C1 cysteine peptidases, as determined by analysis using NCBI Conserved domain database search algorithm. Within the region corresponding to the predicted mature protein, every residue known to be essential for catalytic activity is present in all four sequences [34] (Figure 4). They all have the active-site residues cysteine and histidine, as well as two other residues that play an important role in catalysis. The glutamine which precedes the catalytic cysteine, believed to help in the formation of the oxyanion hole; and an asparagine residue which orients the imidazolium ring of the catalytic histidine. They also have the six conserved cysteine residues which form three disulphide bonds to stabilize the tertiary structure (Figure 4; for TgCP1, TgCP2 and TgCP39 the last cysteine residue is not shown as the corresponding sequence covers the area to which the degenerate PCR primer hybridizes). All four predicted protein sequences possess an incomplete cathepsin pro-peptide inhibitor domain (I29) which is found at the N-terminus of cathepsin L peptidases where it acts as a pro-peptide.

Two of the reported proteins, encoded by TgCP4 and TgCP39, most likely correspond to peptidases detected by the MS analysis, since they matched all identified peptides. The predicted amino acid sequence of the TgCP4 gene matched all three peptides detected for Spot 8, whereas the peptides detected for Spot 5II matched to the predicted amino acid sequence of TgCP39 (Figure 4).

The reported sequences share 59.1–79.1% identity in nucleic acid sequence and 53.3–82.4% identity in amino acid sequence between each other. Interestingly, each of the reported sequences is more related to its *T. vaginalis* homologue than to any of the other reported *T. gallinae* sequences (Table 4).

**Table 4:** Percent identity values between cysteine peptidase genes of *T. gallinae* and their homologues in *T. vaginalis*.

		% identity amino acid sequences								
		TgCP4	TgCP2	TgCP39	TgCP1	TvCP4 (AY679763)	TvCP4 (XM_001310116)	TvCP2 (X77219)	TvCP1 (X77218)	TvCP39 (EU141965)
% identity nucleic acid sequences	TgCP4	100	56.0	82.4	64.2	83.5	83.1	66.3	70.9	83.1
	TgCP2	63.6	100	55.6	53.3	56.4	55.6	69.8	57.1	55.6
	TgCP39	79.1	61.1	100	61.9	85.1	85.1	63.2	71.3	89.7
	TgCP1	64.4	59.1	62.3	100	62.7	61.5	55.0	67.2	63.1
	TvCP4 (AY679763)	83.5	62.8	82.5	64.1	100	96.9	64.7	71.3	92.3
	TvCP4 (XM_001310116)	84.1	63.3	82.8	63.8	97.8	100	64.0	71.6	91.2
	TvCP2 (X77219)	72.2	71.4	67.4	59.6	70.7	70.5	100	60.8	64.0
	TvCP1 (X77218)	74.1	62.9	73.2	70.9	75.4	75.9	67.2	100	72.4
	TvCP39 (EU141965)	83.4	63.0	84.1	64.2	91.6	91.3	70.6	76.3	100

## Discussion

The present study focused on the potential role of molecules secreted by *T. gallinae* in the pathogenesis of this avian parasite. Recently, by analyzing the interaction of *T. gallinae* axenic cultures and their cell-free filtrates with LMH cells, we reported that the damage exerted on the monolayer is a consequence of an interplay between direct and indirect interactions of protozoa and cells [25]. In this study we were able to demonstrate that factors secreted by the parasites were just sufficient to cause the rupture of the monolayer. It is important to emphasize that only secreted products of viable trichomonads were able to destruct the monolayer since the cell-free filtrates from a culture of 10^7^ dead trichomonads had no adverse effect on the monolayer even after 144 hours post incubation. A similar observation was already reported for *T. vaginalis*, a close relative of *T. gallinae* [27]–[30]. Comprehensive research on this human parasite revealed that the action of different cysteine peptidases and other lytic molecules was responsible for cytopathogenic effects of cell-free filtrates on cells as reviewed in Schwebke and Burgess [31].

The present study demonstrated that the cytopathogenic effect of the cell-free filtrate on monolayers is reduced by the addition of the cysteine peptidase inhibitors TLCK and E-64. This indicated that some of the important molecules involved in the monolayer destruction might be cysteine peptidases. TLCK and E-64 have been shown to be excellent inhibitors of all cysteine peptidases produced by *T. vaginalis* [39], [46]. In order to confirm the assumption that peptidases are present in cell-free filtrates, *T. gallinae*-free filtrates with and without peptidase inhibitors were analysed in the 1-D zymograms. The results demonstrated the strong peptidase activity when filtrates without inhibitors were used. In addition, 1-D zymograms demonstrated the difference in the proteolytic activity between cell-free filtrates obtained from different passages of the same axenically grown clonal culture. The cell-free filtrate of the lower passage displayed a much stronger proteolytic activity. This correlated with the data recently presented by Amin et al. [25] that demonstrated a difference in the cytopathogenicity between a low and a high passage of the same *T. gallinae* clonal culture. The cell-free filtrate from the lower passage showed stronger cytopathogenic effects than the higher passage culture. This result indicated the change in the pattern of excretion proteins during *in vitro* cultivation, which could be explained by the change in gene expression of the cultivated parasite. Similar results were observed for *T. vaginalis*, where prolonged *in vitro* cultivation resulted in the loss of virulence and change in the protein expression [47]. Additionally, the attenuation of the culture, as a repercussion of the prolonged *in vitro* cultivation, was already described for *T. gallinae* [24] and an another avian parasite, *Histomonas meleagridis* [48].

One-dimensional zymograms revealed no inhibitory effect of Pepstatin A and PMSF on the proteolytic activity, demonstrating the inertness of these two inhibitors towards peptidases present in the filtrates. However, TLCK and E-64 efficiently inhibited the proteolytic activity of the filtrates. This generally correlated with results obtained with the cell-culture experiments. Furthermore, the results demonstrated that peptidases present in the filtrates, which were blocked by TLCK and E-64, were also partially responsible for the cytotoxicity of the filtrates. However, TLCK, a serine and cysteine peptidase inhibitor did not show the same prominent effect in the cell-culture experiments as in 1-D zymograms. This could be explained as the consequence of the concentration applied in these experiments. In the cell-culture experiments a low concentration (135 µM) of TLCK was used, since with higher concentrations of TLCK toxicity toward LMH cell-line was noticed. Almost a ten-fold higher concentration (1 mM) was used in the 1-D zymogram experiments, which was also the quantity used in many investigations with *T. vaginalis* cysteine peptidases [36], [38], [39], [49]–[51].

Another discrepancy between cell-culture and 1-D zymogram experiments was noticed with the PMSF, a serine peptidase inhibitor. The application of PMSF demonstrated partial inhibition of monolayer destruction, but peptidase activity was not blocked as assayed with the 1-D zymograms. The reason for such a result might lay in the fact that the most abundant type of peptidases present in the filtrates is a cysteine peptidase, which would not be inhibited by PMSF. This would explain that no difference is noticed in 1-D zymograms when samples with and without PMSF were used. However, despite their low concentration, the role of the serine peptidases in the destruction of the monolayer seems to be strong enough, which became obvious in the cell-culture experiments. Finally, the demonstration of Clan CA, family C1, cathepsin L-like cysteine peptidases in the cell-free filtrate of *T. gallinae* by the 2-D substrate SDS-PAGE experiments combined with mass spectrometry supported the hypothesis that cysteine peptidases are the most abundant type of peptidases in the filtrates.

The results of the 2-D substrate SDS-PAGE experiments indicated the presence of at least 7 different peptidases in the cell-free extract of *T. gallinae*. It cannot be excluded that even more peptidases are present in the cell-free extract of *T. gallinae*, since for the 2-D substrate SDS-PAGE analysis, protein samples without peptidase inhibitors were used. This was in particular seen in the mass spectrometric analysis where the lack of material within some analysed protein spots made the identification of further peptides impossible. In such a case the single peptidase would most likely not degrade itself, since it would then be self limiting, but would instead most likely degrade other proteins leading to overall loss of protein in the sample.

The mass spectrometry analysis of the spots from the 2-D SDS-PAGE identified the same peptide, m/z 1466, in five out of six analysed spots. Since most of the spots that share the same peptide have similar molecular weight, but differ slightly in the p*I* value, it is possible that these spots represent isoforms of the same cysteine peptidase. The other explanation of this result would be that these spots indeed represent different proteins. The m/z 1466 peptide, found in most of the proteolytic spots, is common in cysteine peptidases of the related parasite *T. vaginalis* (see Tables 1 and 2). In *T. vaginalis*, many cysteine peptidases that share this peptide also have a similar molecular weight, which resembles the situation presented here. Determination of nearly full genomic sequences of four different cysteine peptidases in *T. gallinae* identified only one cysteine peptidase that possesses this peptide. Even though such a result indicates that the detected spots represent the same protein baring different modifications, the lack of complete genomic sequence of *T. gallinae* leaves the debate open. It should be added that in comparison to proteomics studies analyzing peptidases in *T. vaginalis* [47], [52], in which cell lysates were used for investigation, here, only secreted peptidases were examined. Considering much lower amount of starting material it is no surprise that only single peptides were identified for most of the spots and obtained data did not enable clear distinction whether the analysed spots represent the same or different proteins.

For only two out of six analysed proteolytic spots, more specific cysteine peptidase information according to MS homology search could be found. This result indicated the existence of at least two different cysteine peptidases in the secretome of *T. gallinae*, which was confirmed by identification of nearly complete gene sequences of TgCP4 and TgCP39 that mapped peptides determined by MS analysis. Interestingly, the same approach identified two additional cysteine peptidases genes (TgCP1 and TgCP2), which did not map any of the four peptides. This finding demonstrates a strong conservation in N- and C- terminal sequences between *T. gallinae* cysteine peptidases as well as between *T. gallinae* and *T. vaginalis* enzymes, since degenerated primers used in this PCR approach were based on the conserved N- and C- terminal amino acid sequences of relevant *T. vaginalis* proteins. Comparison of nucleic acid and deduced amino acid sequences between identified *T. gallinae* genes and their homologues in *T. vaginalis* demonstrated that homologous cysteine peptidases from both protozoa are more related to each other than analysed enzymes from a single protozoan. Such result could indicate that different cysteine peptidases evolved at least at the same time as the speciation of trichomonads.

The finding of cysteine peptidases in the cell-free filtrate of *T. gallinae* is in agreement with several investigations on related parasites like *T. vaginalis* and *T. foetus*. The work on *T. vaginalis* and *T. foetus* demonstrated the involvement of cysteine peptidases in host tissue invasion and destruction by the parasites [29], [38], [53], [54]. In particular *T. vaginalis* cysteine peptidases found extracellularly, were shown to be involved in the degradation of the mucin layer that covers epithelial cells [41] and in the apoptosis of human vaginal epithelial cells [29], indicating an important role in the virulence of this parasite.

Conclusively, the present study had shown that the proteins secreted by *T. gallinae* possessed the proteolytic activity which contributed to the detachment of the monolayer. For the first time molecular characterization involving 1-D and 2-D zymograms in combination with mass spectrometric analysis showed that the cysteine peptidases are present in the pool of proteins secreted by *T. gallinae*. Finally, further demonstration that cysteine peptidases take part in the cytopathogenic effects of *T. gallinae* makes these proteins excellent candidates for virulence factors.

## Materials and Methods

### Tested clonal culture

Cell-free filtrates of the *T. gallinae* axenic clonal culture named *Trichomonas gallinae* /Budgerigar/Austria/5895-C1/06 (shortly, clone 5895-C1/06) were investigated. The name assignment reflects the species of bird/country of origin/diagnostic number-clone number/year of isolation. In the present study a distinction between lower (P18, P49, P53) and high passages (P130) of the same clonal culture was made.

### Cell culture

Permanent chicken liver cells (LMH; ATCC® Number: CRL-2117™) were grown in Medium 199 + Earle's salts + L-Glutamine (Invitrogen/Gibco, Paisley, U.K.). The medium was supplemented with 10% of FBS, 10% Tryptose Phosphate broth, penicillin (40,000 IU/ml) and streptomycin (40 mg/ml) (all Invitrogen/Gibco, Paisley, U.K). One millilitre of the media suspension containing 1×10^6^ cells was inoculated into 75 cm^2^ flasks with filtered caps (Sarstedt, Wiener Neudorf, Austria) containing 6 ml medium. Cells were kept in a controlled atmosphere of 5% CO_2_ at 37 °C and around 85–90% humidity. After 72 h of incubation, a confluent monolayer of LMH cells was obtained with an average of 7×10^6^ cells per flask. Cells were passaged every three to four days depending on their density.

### Preparation of cell-free filtrate from axenic *T. gallinae* culture

For the analysis on LMH monolayers the cell-free filtrate was prepared in the following way: 10^7^ cells of a clonal culture of *T. gallinae* were incubated at 37°C for 24 h in the same medium as used for growth of LMH. Trichomonad culture was centrifuged at 3300×g for 5 min and then the supernatant from the culture was filtered through 0.22 μm cellulose acetate filters (Millipore, VWR).

For analysis in one- (1-D) and two-dimensional (2-D) zymogram and conventional SDS PAGE the filtrate was prepared in the following way: *T. gallinae* clonal culture was grown in Hollander fluid (HF) medium as described recently [55]. After 24 h of incubation, the trichomonad culture was centrifuged at 3300×g for 5 min; the pellet was washed three times in phosphate buffer saline (PBS, pH 7.2) and was then suspended in 1 ml HF medium without serum. Motile trichomonads were counted by a Neubauer cell counting chamber (Reichert, Buffalo, NY). The concentration of the inoculum was adjusted to 10^7^ motile trophozoites. 10^7^ trichomonad cells were grown for 24 h in 10 ml HF medium without serum and the cell-free filtrate was prepared. The trichomonad culture was centrifuged at 3300×g for 5 min, and the supernatant was filtered through 0.22 μm cellulose acetate filters (Millipore, VWR). Afterwards, 1 mM PMSF, 5 μM of Pepstatin A, 1 mM TLCK and 270 μM E-64 were added to the cell-free filtrate and incubated for 30 min on ice before concentrating the proteins. Two types of filters were used to concentrate the proteins of the cell-free filtrate. Firstly, CENTRIPREP® Centrifugal Filter Device 3K (Millipore, Vienna, Austria) was applied to reduce the initial volume of the filtrate (15 ml) to 500 μl. After that, the samples were more concentrated using Amicon® Ultra−0.5 3K centrifugal filter device (Millipore, Vienna, Austria) to obtain a final volume of 100 μl. Both types of filters were used following manufacturer's instructions. With the samples for 2-dimensional (2-D) sodium dodecyl sulphate (SDS)-polyacrylamide gel electrophoresis (PAGE) buffer was exchanged (three times) to 10 mM Tris-Cl pH 7.2 by using the same Amicon® Ultra−0.5 3K centrifugal filter device (Millipore, Vienna, Austria). For all samples the protein concentration was measured by Bradford Protein Assay (Fermentas, Thermo Scientific).

### Assessment of LMH monolayer

Each monolayer was investigated visually by an inverted light microscope to detect the effect of the cell-free filtrates on LMH monolayer. Assessment of the monolayer was performed as described recently [25].

### Effect of peptidase inhibitors on the cell-free filtrate

Firstly, preliminary experiments were done to establish the concentration of each peptidase inhibitor that was not toxic to the monolayer cell culture. In these initial experiments different concentrations of the following peptidase inhibitors were added to not-infected monolayer: Phenylmethylsulfonyl fluoride (PMSF) as serine peptidase inhibitor (0.2 mM, 0.5 mM, 1 mM), Pepstatin A as aspartic peptidase inhibitor (1 μM, 2.5 μM, 5 μM), N-α-p-Tosyl-L-Lysine chloromethyl ketone (TLCK) as cysteine and some serine peptidase inhibitor (135 μM, 540 μM, 1 mM), and L-3-carboxyl-2,3-trans-epoxysuccinyl-leucylamido (4-guanidino) butane (E-64) as cysteine peptidase inhibitor (70 μM 140 μM, 270 μM), all purchased from Sigma-Aldrich, Steinheim, Germany.

### Cytotoxicity assay

CellTiter 96® aqueous one solution cell proliferation assay (Promega Corporation, Madison, USA) was used following manufactureŕs instructions to investigate the cytotoxicity of the cell-free filtrate on cell culture. The cell-free filtrate from 10^7^ trichomonad cells was prepared as described in chapter 2.3. and 270 μM of E-64 was added to cell-free filtrate before its incubation with the monolayer. Cytotoxicity assay was performed as described recently [25].

### One- and two dimensional gels electrophoresis and zymographic analysis

For one dimension (1-D) zymographic analysis, 24 µg of concentrated proteins from cell-free filtrates with and without peptidase inhibitors were separated by discontinuous SDS-PAGE (8%) copolymerized with 0.2% gelatin as substrate. In parallel, the same samples were investigated by conventional 1-D SDS-PAGE (8%). After electrophoresis, the substrate gel was incubated in 2.5% Triton X-100 (Bio-Rad Laboratories GmbH, Vienna, Austria) for 1 h at room temperature to remove SDS. Subsequently the peptidases were activated by incubating the gel in zymogram development buffer (Bio-Rad Laboratories GmbH, Vienna, Austria) for 24 h at 37°C. All gels were stained with PageBlue^™^ Protein Staining Solution (Fermentas, Fisher Scientific Österreich, Vienna, Austria) and clear bands in gelatin gels were indicative of proteolytic activity. All experiments were repeated twice.

For 2-D zymographic analysis of the concentrated proteins from cell-free filtrate the buffer was exchanged to rehydration buffer (8M Urea (GE Healthcare, UK), 2% CHAPS (Sigma-Aldrich, Steinheim, Germany), 50 mM DTT (Sigma-Aldrich, Steinheim, Germany), 0.2% Servalyt®-Ampholytes pH 3–5 (Serva), trace bromphenol blue (Sigma-Aldrich, Steinheim, Germany) via Zeba™ Spin Desalting Columns, 7K MWCO (Pierce, Fisher Scientific Österreich, Vienna, Austria) according to manufacturer's instructions. For first-dimension of both silver stained and PageBlue^™^ stained zymogram gels, 240 µg protein was applied to IPG-strip (7 cm, pH 3–6, Bio-Rad Laboratories GmbH, Vienna, Austria) by passive in-gel rehydration (16 h at room temperature). Isoelectric focusing (IEF) was carried out in a Protean IEF cell (Bio-Rad Laboratories GmbH, Vienna, Austria). Voltage was increased in the slow ramp mode; 100 V in 1 h, 250 V in 1 h, 500 V in 1 hour, 1000 V in 1.5 h, 4000 V in 1 h followed by focusing at 4000 V until 18 000 Vhs were reached. For both types of analyses the IPG strips were equilibrated before the second dimensional run first in 6M Urea (GE Healthcare, UK), 2% SDS (Merck, VWR), 0.375M Tris-Cl (Merck, VWR) 20% glycerol (Sigma-Aldrich, Steinheim, Germany) pH 8.8 containing 2% DTT (Sigma-Aldrich, Steinheim, Germany) and afterwards in the same buffer supplemented with 2.5% iodoacetamide (Sigma-Aldrich) for 10 min at room temperature each. In the second-dimension proteins were resolved by SDS-PAGE (8%) and substrate (0.2% gelatin) SDS-PAGE (8%) for silver-stained gels and zymograms, respectively. Silver-staining was performed according to Shevchenko et al. [56]. Zymogram gels were treated and stained as described for 1-D zymograms gels. Clear spots were indicative for proteolytic activity. Images of all gels (1-D and 2-D) were taken with Molecular Imager ChemiDoc™ XRS System (Bio-Rad Laboratories GmbH, Vienna, Austria) using Quantity One v4.6.3 software (Bio-Rad Laboratories GmbH, Vienna, Austria).

### Protein digestion, peptide extraction and mass spectrometric analysis

Selected spots from 2-D gels were excised, washed, destained, reduced with DTT (Sigma-Aldrich, Steinheim, Germany) and alkylated with Iodoacetamide (Sigma- Aldrich, Steinheim, Germany). The in-gel digest was carried out with trypsin (Trypsin Gold, Mass Spectrometry Grade, Promega Corporation, Madison, USA) [56] and after extraction the dried peptides were de-salted using µZip-Tips C18 (Millipore, VWR, Vienna, Austria) according to the manufacturer's instructions. De-salted peptides (0.5 µl) were spotted onto a pre-spotted AnchorChip MALDI (PAC target, Bruker Daltonik GmbH, Leipzig, Germany).

Data were acquired on a Matrix Assisted Laser Desorption Ionisation Tandem Time-of-Flight (MALDI-TOF/TOF) mass spectrometer (Ultraflex II, Bruker Daltonik GmbH, Leipzig, Germany) in MS and MS/MS modes. Spectra processing and peak annotation were carried out using FlexAnalysis and Biotools (Bruker Daltonik GmbH, Leipzig, Germany).

### MS data analysis and de novo peptide sequencing

Processed spectra were searched via Mascot in the Swiss-Prot database (release 56.5) or in NCBInr (20111005) using the following search parameters: taxonomy Trichomonas; global modifications carbamido-methylation on cysteine; variable modifications oxidation on methionine; MS tolerance 100 ppm; MS/MS tolerance 1 Da; one missed cleavage allowed. Identifications were considered statistically significant where p <0.05.

Peptide de Novo sequencing was carried out manually using FlexAnalysis. These sequences were then used for a homology search using MS Homology in ProteinProspector 5.9.0. http://prospector.ucsf.edu/prospector/cgi-bin/msform.cgi?form=mshomology. The searched database was UniProtKB.2011.01.11 with following parameters: taxonomy search in *Trichomonas vaginalis*, 0.5 kDa mass tolerance, no enzyme digest, one missed cleavage allowed, minimal match of peptides 1 and BLOSUM62 as score matrix.

### PCR amplification and sequence analysis

The cysteine peptidase sequences were amplified from the genomic DNA of clone 5895-C1/06 by PCR with degenerate primers. Genomic DNA from clone 5895-C1/06 was prepared using the DNeasy Blood and Tissue Kit (Qiagen, Vienna, Austria) following the protocol for purification of total DNA from cultured animal cells according to the manufactureŕs instructions. Two different sets of primers were used for PCR. The design of both primer sets was based on the N- and C- terminal conserved amino acid regions, deduced from the alignment of different *T. vaginalis* cysteine peptdeases. First primer set TgCPsp8F (5′-atg tty gtn car gch cay gar car aar gcn tt-3′)/TgCPsp8R (5′-tt rtc ytg ngg dat rca ngc cat ngt ngc-3′) was based on the MFVQAHEQKAF and ATMACIPQDK peptides, respectively. The second primer set TgCPsp5F (aay atg tty acn ggn gay gar ta-3′)/TgCPsp5R (5′-gc ytc ncc rca ytg rtt rtt ytt-3′) was based on NMFTGDE and KNNQCGEA peptides, respectively. All primers were synthesized by Eurofins MWG Operon Ebersberg, Germany.

Hot start procedures were used for PCR amplification using the “HotStarTaq Master Mix Kit” (Qiagen, Vienna, Austria). PCR was carried out in a 25-μl reaction mixture by using 100ng of trichomonad DNA and each of the primers at 1µM final concentration. The reaction mixture was subjected to an initial denaturation at 95°C for 15 minutes, followed by 40 cycles of 94°C for 30 seconds, 51.4°C (for reaction with TgCPsp5F/ TgCPsp5R) or 53.9°C (for reaction with TgCPsp8F/ TgCPsp8R) for 30 seconds and 72°C for 1.5 minutes + 6 seconds/cycle, ending at 72^°^C for 10 minutes. Amplification products (25 µl) were electrophoresed in a 1.0% Tris acetate-EDTA-agarose gel for 60 minutes at 100 V. The gels were stained with ethidium bromide, visualized under UV light (Bio-Rad Universal Hood II, Bio-Rad Laboratories, California, USA), 900bp fragments were excised and then purified with the QIAquick® Gel Extraction Kit (Qiagen, Vienna, Austria).

Prior to sequencing the PCR fragments were cloned to pCR®4-TOPO vector by using TOPO TA Cloning® Kit for sequencing (Invitrogen, Austria). The clones were checked for the presence of a PCR fragment by digestion with Eco*RI* restriction enzyme (Invitrogen, Austria) and positive clones were sequenced in both directions with custom primers M13–21F and M13–29R by LGC Genomic (Berlin, Germany).

Assembly and analyses of DNA sequences as well as alignments of both nucleotide and amino acid sequences were performed with Accelrys Gene, version 2.5 (Accelrys, San Diego, CA) and Lasergene (DNASTAR Inc.) software packages. The DNA sequence of TgCP4 was obtained with PCR using TgCPsp8F/TgCPsp8R as primer pair, whereas sequences of TgCP1, TgCP2, and TgCP39 were obtained by PCR with the TgCPsp5F/TgCPsp5R primer pair. GenBank^TM^ database searches of obtained sequences were carried out with BlastN, BlastP and specialised BLASTs for conserved domains and conserved domain architecture with default settings. All four sequences were deposited in EMBL database and their accession numbers are HE797913-HE797016.

## Supporting Information

Figure S1 **The MS spectra of all analysed spots.** Peaks of charged peptides that were further analysed are circled. Data were acquired on a Matrix Assisted Laser Desorption Ionisation Tandem Time-of-Flight (MALDI-TOF/TOF) mass spectrometer (Ultraflex II, Bruker Daltonik GmbH, Leipzig, Germany) in MS and MS/MS modes. Spectra processing and peak annotation were carried out using FlexAnalysis and Biotools (Bruker Daltonik GmbH, Leipzig, Germany).
(PPTX)

Figure S2 **MS/MS spectra of charged peptides m/z 1121.4, m/z 1466.6, m/z 850.5 from spot 8.** Peptide de Novo sequencing was carried out manually using FlexAnalysis.
(PPTX)

Figure S3 **MS/MS spectra of charged peptides m/z 1879.8, m/z 850.5 from spot 5II.** Peptide de Novo sequencing was carried out manually using FlexAnalysis.
(PPTX)
