# Supplementary figures and images for: Cysteine Peptidases, Secreted by Trichomonas gallinae, Are Involved in the Cytopathogenic Effects on a Permanent Chicken Liver Cell Culture
Source: PLoS One. 2012 May 23;7(5):e37417. doi: 10.1371/journal.pone.0037417 (PMC3359344; doi:10.1371/journal.pone.0037417)

## Slide 1
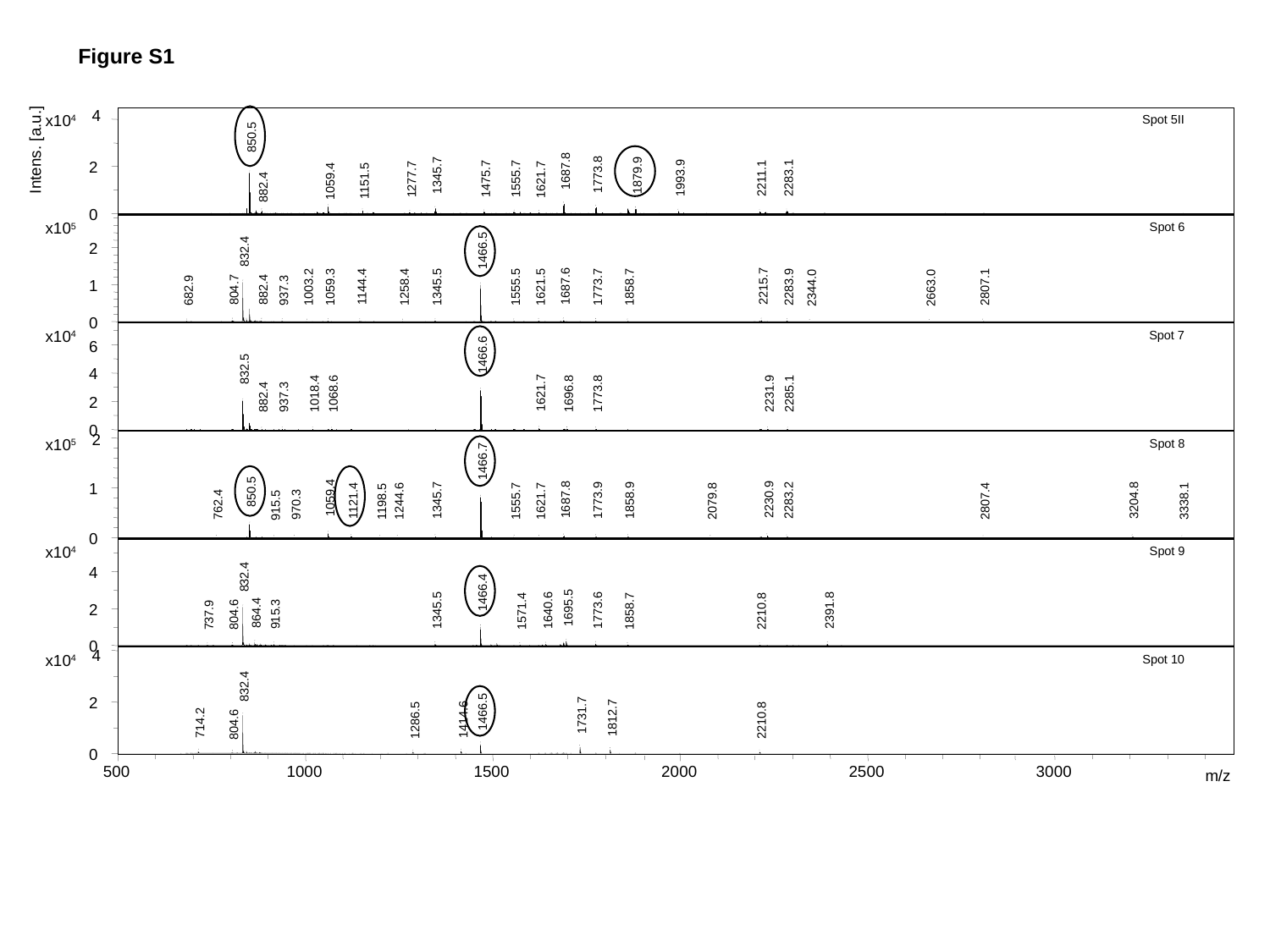

Figure S1
4
x104
Spot 5II
850.5
Intens. [a.u.]
2
1687.8
1773.8
1345.7
1879.9
2283.1
1993.9
2211.1
1475.7
1555.7
1277.7
1621.7
1059.4
1151.5
882.4
0
x105
Spot 6
2
1466.5
832.4
1
1687.6
2215.7
1059.3
1144.4
1345.5
1621.5
1773.7
2283.9
1003.2
1258.4
1555.5
1858.7
2807.1
2344.0
2663.0
804.7
882.4
937.3
682.9
0
x104
Spot 7
6
1466.6
832.5
4
1621.7
1696.8
1018.4
1773.8
2231.9
1068.6
2285.1
882.4
937.3
2
0
Spot 8
1466.7
850.5
1059.4
2230.9
1687.8
1773.9
1858.9
1121.4
1345.7
2283.2
3204.8
1555.7
1621.7
2079.8
1198.5
1244.6
2807.4
3338.1
762.4
970.3
915.5
0
2
x105
1
x104
Spot 9
4
832.4
1466.4
2
1695.5
1773.6
1345.5
2391.8
1640.6
1571.4
1858.7
2210.8
864.4
915.3
804.6
737.9
0
4
x104
Spot 10
832.4
2
1466.5
1731.7
1812.7
1414.6
2210.8
1286.5
714.2
804.6
0
500
1000
1500
2000
2500
3000
m/z

Supplement: Figure S1 — The MS spectra of all analysed spots. Peaks of charged peptides that were further analysed are circled. Data were acquired on a Matrix Assisted Laser Desorption Ionisation Tandem Time-of-Flight (MALDI-TOF/TOF) mass spectrometer (Ultraflex II, Bruker Daltonik GmbH, Leipzig, Germany) in MS and MS/MS modes. Spectra processing and peak annotation were carried out using FlexAnalysis and Biotools (Bruker Daltonik GmbH, Leipzig, Germany). (PPTX) [file pone.0037417.s001.pptx]

## Slide 1
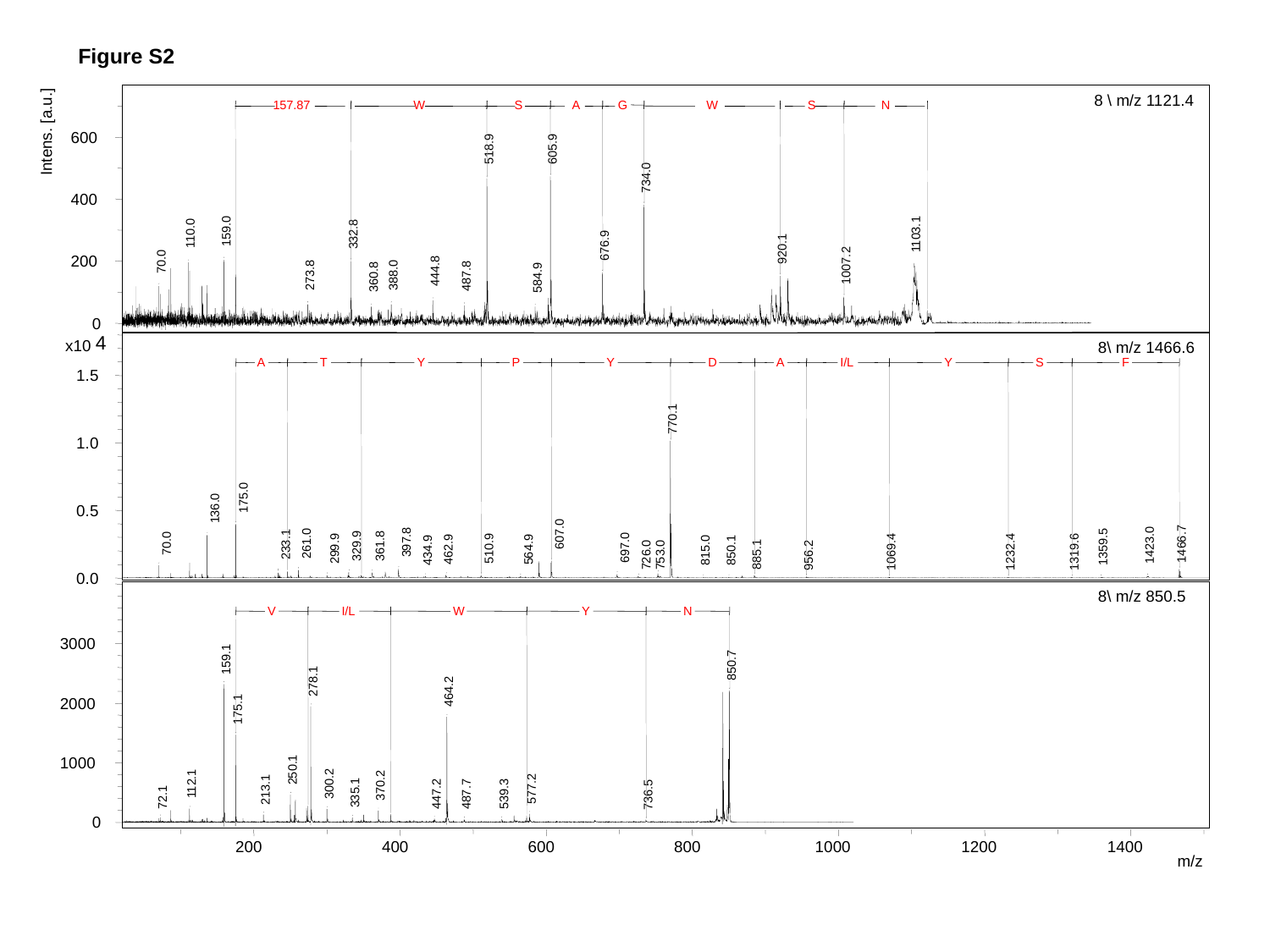

Figure S2
8 \ m/z 1121.4
157.87
W
S
A
G
W
S
N
Intens. [a.u.]
600
518.9
605.9
734.0
400
159.0
110.0
332.8
1103.1
676.9
920.1
200
70.0
1007.2
444.8
273.8
388.0
487.8
360.8
584.9
0
A
T
Y
P
Y
770.1
175.0
136.0
607.0
397.8
70.0
261.0
1466.7
233.1
1423.0
329.9
361.8
1359.5
697.0
510.9
299.9
462.9
564.9
434.9
815.0
850.1
1069.4
1232.4
1319.6
885.1
726.0
753.0
956.2
4
x10
 8\ m/z 1466.6
D
A
I/L
Y
S
F
1.5
1.0
0.5
0.0
 8\ m/z 850.5
V
I/L
W
Y
N
3000
159.1
850.7
278.1
464.2
2000
175.1
1000
250.1
112.1
300.2
370.2
577.2
213.1
335.1
447.2
539.3
487.7
736.5
72.1
0
200
400
600
800
1000
1200
1400
m/z

Supplement: Figure S2 — MS/MS spectra of charged peptides m/z 1121.4, m/z 1466.6, m/z 850.5 from spot 8. Peptide de Novo sequencing was carried out manually using FlexAnalysis. (PPTX) [file pone.0037417.s002.pptx]

## Slide 1
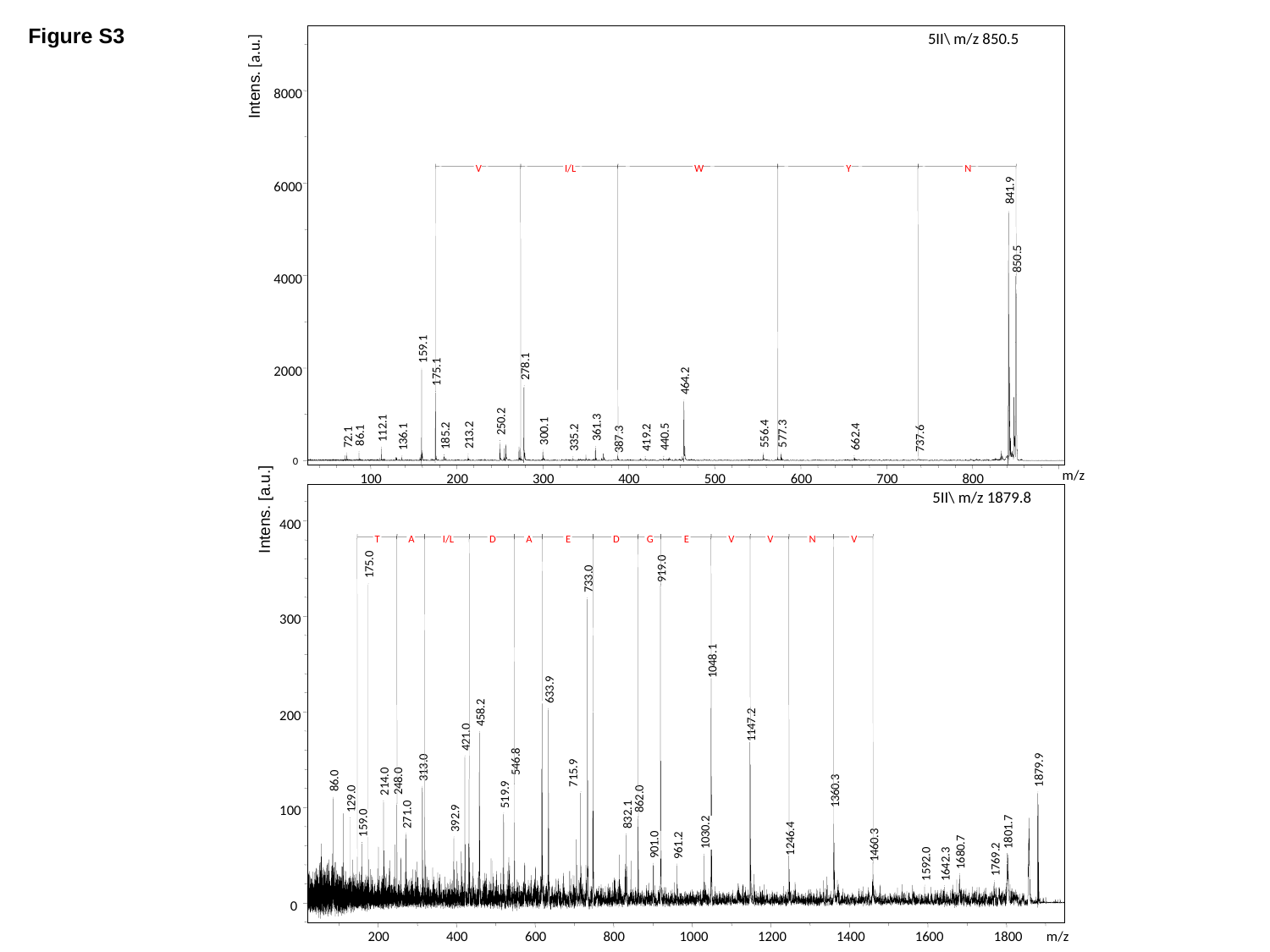

Figure S3
5II\ m/z 850.5
Intens. [a.u.]
8000
V
I/L
W
Y
N
6000
841.9
850.5
4000
159.1
278.1
2000
175.1
464.2
250.2
361.3
112.1
300.1
577.3
556.4
86.1
213.2
185.2
662.4
440.5
136.1
72.1
335.2
419.2
737.6
387.3
0
m/z
100
200
300
400
500
600
700
800
 5II\ m/z 1879.8
Intens. [a.u.]
400
T
A
I/L
D
A
E
D
G
E
V
V
N
V
175.0
919.0
733.0
300
1048.1
633.9
458.2
200
1147.2
421.0
546.8
313.0
1879.9
715.9
86.0
248.0
214.0
1360.3
519.9
129.0
862.0
100
271.0
832.1
392.9
159.0
1801.7
1030.2
1246.4
901.0
1460.3
961.2
1680.7
1769.2
1592.0
1642.3
0
200
400
600
800
1000
1200
1400
1600
1800
m/z

Supplement: Figure S3 — MS/MS spectra of charged peptides m/z 1879.8, m/z 850.5 from spot 5II. Peptide de Novo sequencing was carried out manually using FlexAnalysis. (PPTX) [file pone.0037417.s003.pptx]
